# Association of Metabolic Syndrome with Decreased Glomerular Filtration Rate among 75,468 Chinese Adults: A Cross-Sectional Study

**DOI:** 10.1371/journal.pone.0113450

**Published:** 2014-11-21

**Authors:** Hui Song, Xiuying Wang, Qingqing Cai, Weijie Ding, Shuiping Huang, Lang Zhuo

**Affiliations:** 1 Department of Epidemiology, School of Public Health, Xuzhou Medical College, Xuzhou, Jiangsu, China; 2 Division of Nephrology, Xuzhou Central Hospital, Xuzhou, Jiangsu, China; National Centre for Scientific Research “Demokritos”, Greece

## Abstract

**Background:**

The impact of the various elements of metabolic syndrome (MetS) on chronic kidney disease (CKD) has been conflicting. Therefore, in the present study we aimed to examine the association of MetS and its components with decreased glomerular filtration rate (GFR).

**Methods:**

A total of 75,468 urban workers who underwent annual health examinations under the auspices of the local governments between March 2010 and September 2012 at the health examination center of Xuzhou center hospital were enrolled in the cross-sectional survey. Decreased GFR was defined as an estimated GFR <60 mL/min per 1.73 m^2^. The definition of MetS was based on the most-recent interim joint consensus definition, requiring any three of the five components, consisting of elevated blood pressure (BP), fasting plasma glucose (FPG), or triglycerides (TG), reduced high density lipoprotein-cholesterol (HDL-c), and obesity.

**Results:**

MetS was related to the reduced GFR with an odds ratio [95% confidence interval (CI)] of 1.43 (1.13–1.83). In multivariable analyses, individual components of MetS that were independently associated with decreased GFR were elevated BP (OR 1.34, 95% CI 1.00–1.78), low HDL-c (OR 1.88, 95% CI 1.44–2.43), and elevated FPG (OR 1.42, 95% CI 1.09–1.85). The age-adjusted population-attributable risk percent (PARP) for reduced GFR was 27.55%, 19.67% and14.31% for elevated BP, low HDL-c and elevated FPG respectively. The multivariate-adjusted odds ratios (95% CI) of decreased GFR were 1.70(1.11–2.60), 2.38(1.53–3.71), or 4.11(2.42–6.98) for those with 1, 2, or 3 critical elements (versus zero), respectively. The corresponding multivariate-adjusted odds ratios (95% CI) of decreased GFR were 1.11(0.84–1.48) and 0.89(0.63–1.27) for those with 1 or 2 noncritical components (versus zero), respectively.

**Conclusions:**

We concluded that various elements of MetS and the cumulative number of MetS should not be considered indiscriminately as risk factors for reduced GFR.

## Introduction

Chronic kidney disease (CKD) has received increased attention as a major public health issue worldwide. Until recently, the population prevalence of CKD has exceeded 10%, more than 50% of whom were in high-risk sub-populations [1]. Moreover, global deaths from kidney disease have risen by 83% since 1990 [2]. A better understanding of the nature of CKD, its risk factors, and implementation of prevention strategies are, therefore, keys to saving many lives [3].

Subsequent articles suggested that hypertension and diabetes were deemed to be the leading causes of CKD in all developed and many developing countries [4]–[6]. Increasingly, the important contribution of metabolic syndrome (MetS) in CKD was recognized, which helped to obtain a further understanding of the aetiology of CKD. Epidemiologic observation suggested an independent association for MetS and CKD [7], [8]. However, studies on the association between various elements of MetS and CKD, to some extent, were conflicting. For example, in a cross-sectional study of 15,160 Chinese adults aged 35–74 years, Chen et al [9] reported a significant association of CKD with low high density lipoprotein-cholesterol (HDL-c), elevated fasting plasma glucose (FPG) and abdominal obesity. Nevertheless, Kitiyakara et al [10] found that only high blood pressure (BP) was associated with the prevalence of CKD among all five MetS components in a Southeast Asian cohort. Meanwhile, it was well established that the odds ratio of CKD was proportional to the cumulative number of MetS components [11]. However, the association between CKD and the number of critical or noncritical components of MetS was not well known. Thus, further supporting evidence is needed.

In the present study, our objective was to evaluate the association of MetS with decreased GFR in a large cross-sectional study among 75,468 Chinese adults. The ad hoc objectives were to examine the relationship between individual elements of MetS and decreased GFR, and to determine if there was a biologic gradient in the association between the odds of decreased GFR and the number of critical or noncritical components of MetS.

## Material and Methods

### Study population

The data for this retrospective study was an electronic record of urban workers who underwent annual health examinations under the auspices of the local governments between March 2010 and September 2012 at the health examination center of Xuzhou Center Hospital, the affiliated hospital of Xuzhou Medical College in Xuzhou, Jiangsu, China. The electronic record form consisted of four main parts, namely, participants registration files (register code, name, age, sex, office address, and register date), anthropometric measurements files (body height, body weight, systolic BP, and diastolic BP), laboratory files (blood and urine tests results), and other files (personal history of chronic disease, cardiovascular disease, renal transplant or dialysis, and medication history). After excluding participants with a renal transplant or on dialysis, a history of established cardiovascular disease, or those without related information (serum creatinine and all parameters related to MetS), data from 75,468 study subjects aged 18 years or older were used in our analysis. The ethics committee of Xuzhou Medical College of China approved this study, and the participants provided written informed consent.

### Study measurements

Demographic, anthropometric, and laboratory data were collected from all participants. Information regarding age, gender, and medical history was obtained. Height, body weight, and blood pressures were included in the physical examinations, in which BP was measured 3 times by professional nurses using a random-zero sphygmomanometer after a 5-minute sitting, and their averages were recorded. Blood specimens were drawn after a 12 h overnight fast for examining lipids, glucose, and serum creatinine. Triglycerides (TG) levels were analyzed enzymatically using commercially available reagents. HDL-c was determined by immunoturbidimetry. FPG was measured using a modified hexokinase enzymatic method. Serum creatinine(Cr) was measured with Jaffe's kinetic method. Body mass index (BMI) was computed as weight [Kg]/(height[m])^2^.

### Diagnosis of metabolic syndrome

The definition of MetS in our study was modeled after the Adult Treatment Panel III (ATP III) as the presence of three or more of the following risk factors [12]: 1) Obesity: BMI ≥25 kg/m^2^, 2) Elevated TG: Serum TG level ≥1.70 mmol/L (150 mg/dl), 3)Reduced HDL-c: HDL-c level <1.04 mmol/L (40 mg/dl) in men or <1.30 mmol/L (50 mg/dl) in women, 4) Elevated BP: BP ≥130/85 mmHg and/or use of antihypertensive medications, and 5) Elevated FPG: Serum glucose level ≥6.11 mmol/L (110 mg/dl) and/or use of insulin or hypoglycemic medication. BMI was used in our definition as the data set did not have enough measurements of waist circumference. BMI ≥25 Kg/m^2^ was proposed as a cutoff for the diagnosis of obesity in Asians [13].

### Definition of decreased GFR

The estimated GFR (eGFR), as an indicator of kidney function, was calculated using a formula developed by adaptation of the Modification of the Diet Renal Disease (MDRD) equation on the basis of data from Chinese chronic kidney disease patients [14]: eGFR (mL/min/1.73 m^2^)  = 175× (serum Cr)^−1.234^× (age)^−0.179^×0.79 (if female). Serum Cr: serum creatinine (mg/dl), 1 mg/dl = 88.4 umol/L. Decreased GFR was defined as an eGFR <60 mL/min/1.73 m^2^.

### Statistical analysis

Statistical analyses were conducted by SPSS 18.0 for Windows. Baseline characteristics were compared between participants with and without MetS. Continuous variables were expressed as mean ± SD and compared using a Student's t test. Categorical variables were described as proportions and compared by the chi-square test. Curve diagrams for the prevalence of decreased GFR were drawn according to age and gender. The prevalence of decreased GFR for participants was calculated by the status (absence and presence) of each of the five MetS components, as well as the number of MetS components present.

To explore the association of individual MetS components with decreased GFR, odds ratio (OR) and 95% confidence interval (CI) were first estimated in univariate logistic regression models. Age and sex adjusted odds ratios of decreased GFR were calculated in multivariable logistic regression to circumvent the potential confounders (age and sex). We then constructed multivariable logistic regression models by including all five MetS elements along with age and gender to assess the independent effect of each element. Age-adjusted population-attributable risk percent (PARP) of critical components of MetS was calculated using the following formula [15]. 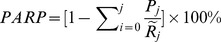



*P_j_* and 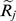 represents the proportion of all cases and relative risk in stratum j respectively.

The association between the cumulative number of MetS components and decreased GFR was first examined with univariate logistic regression models and then examined with multivariable logistic regression models adjusting for age and gender. Later, all five MetS components were divided into critical components arm and noncritical components arm. OR and 95% CI of reduced GFR were calculated across MetS elements' categories after adjusting for age and gender. All tests were two sided with *P*<0.05 considered statistically significant.

## Results

### Baseline characteristics

Eligible data from 75,468 subjects were used. Mean age was 48.79±13.76 yr with 41.1% youth (18–44 yr), 36.8% middle-aged (45–59 yr), and 22.1% elderly (60- yr). There was 56.3% (n = 42,488) male participants. The percentages of elevated BP, elevated TG, reduced HDL-c, elevated FPG, and obesity were 51.3%, 30.2%, 29.9%, 14.8%, and 44.2%, respectively. Of the subjects, 21,497 (28.5%) subjects met criteria for the MetS, and 273 (0.4%) had decreased GFR.

### General characteristics of study participants by the presence of MetS

Table 1 presents the general characteristics of all participants by MetS status. Participants with MetS had a higher mean age, as well as a higher proportion of males (*P*<0.001). The mean levels of SBP, DBP, TG, HDL-c, FPG, and BMI were significantly different between the two groups (*P*<0.001). Furthermore, eGFR was slightly lower for those with MetS (*P*<0.001).

**Table 1:** Baseline characteristics of study participants by presence of the metabolic syndrome.

Characteristics	Metabolic Syndrome Present (n = 21497)	Metabolic Syndrome Absent (n = 53971)	*P*- value
Age (years)	52.32±13.17	47.39±13.73	<0.001
Men	14264 (66.35)	28249 (52.34)	<0.001
Body height (cm)	166.54±8.97	165.61±8.39	<0.001
Body weight (kg)	76.17±12.04	65.00±11.16	<0.001
Body mass index (kg/m^2^)	27.36±2.73	23.61±2.94	<0.001
Systolic blood pressure(mm Hg)	138.73±15.69	123.07±16.30	<0.001
Diastolic blood pressure(mm Hg)	87.22±10.27	78.06±10.36	<0.001
Triglyceride (mmol/L)	2.60±2.06	1.18±0.81	<0.001
Fasting plasma glucose (mmol/L)	6.24±1.84	5.31±0.91	<0.001
HDL-cholesterol (mmol/L)	1.11±0.25	1.42±0.32	<0.001
eGFR (ml/min/1.73 m^2^)	134.23±34.69	137.97±31.95	<0.001

Data are n (%) or mean ± SD; HDL, high-density lipoprotein. eGFR, estimated glomerular filtration rate.

### Prevalence of decreased GFR stratified by age and gender

**Figure 1:**
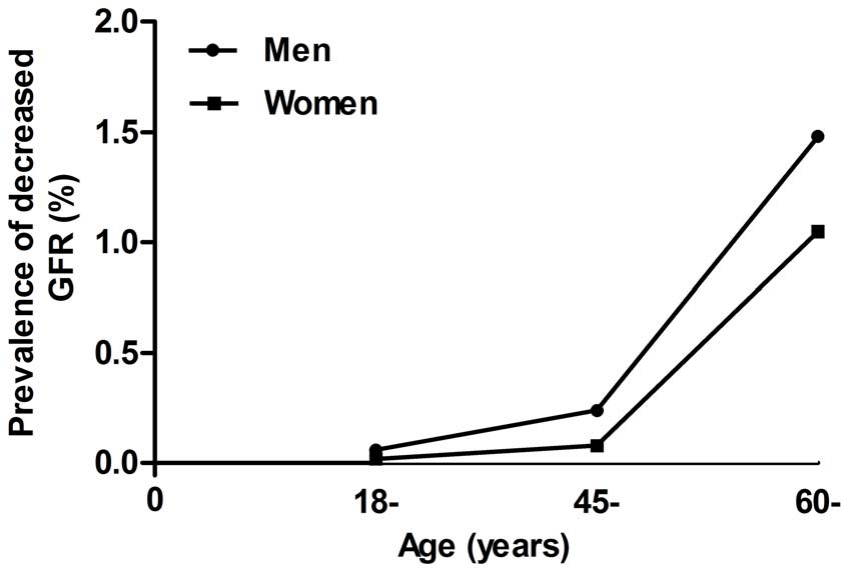
of decreased GFR by age and gender. GFR, glomerular filtration rate. Decreased GFR was defined as an estimated glomerular filtration rate <60 ml/min per 1.73 m^2^ according to a formula developed by adaptation of the Modification of the Diet Renal Disease (MDRD) equation on the basis of data from Chinese chronic kidney disease patients.

Figure 1 depicts the prevalence of decreased GFR stratified by age and gender. The prevalence of decreased GFR increased with age in both men and women. Men had a higher prevalence of reduced GFR than women (0.42% vs 0.29%, *P* = 0.005).

### Association between individual components of MetS and decreased GFR

In univariate analyses, each component of MetS was associated with decreased GFR. After adjusted by age and gender, most results appeared consistent except obesity (Table 2). In multivariable analyses for individual MetS components (Table 3), there was a significant association between elevated BP, reduced HDL-c, elevated FPG and decreased GFR, with estimated ORs of 1.34(1.00–1.78), 1.88(1.44–2.43) and 1.42(1.09–1.85), respectively; the other 2 components seemed to be noncritical factors. The age-adjusted population-attributable risk percent (PARP) of elevated BP, reduced HDL-c, and elevated FPG for reduced GFR was 27.55%, 19.67%, and 14.31%, respectively.

**Table 2:** Prevalence and odds ratio of decreased GFR among participants with and without components of the metabolic syndrome.

Variables	N	Prevalence (%)	OR (95% CI)	
			Unadjusted	Age and Sex Adjusted
Metabolic syndrome				
No	53971	0.28	1.00	1.00
Yes	21497	0.57	2.03 (1.60–2.58)	1.43 (1.13–1.83)
Elevated blood pressure				
No	36739	0.19	1.00	1.00
Yes	38729	0.53	2.87 (2.18–3.78)	1.37 (1.03–1.82)
Elevated triglyceride				
No	52683	0.32	1.00	1.00
Yes	22785	0.46	1.45 (1.13–1.85)	1.43 (1.12–1.82)
Reduced HDL-cholesterol				
No	52936	0.28	1.00	1.00
Yes	22532	0.55	1.93 (1.52–2.45)	1.93 (1.51–2.47)
Elevated fasting glucose				
No	64263	0.29	1.00	1.00
Yes	11205	0.78	2.70 (2.09–3.48)	1.51 (1.16–1.95)
Obesity				
No	42095	0.31	1.00	1.00
Yes	33373	0.42	1.34 (1.05–1.69)	0.97 (0.76–1.23)
No. of Components				
0	17348	0.10	1.00	1.00
1	19008	0.25	2.38 (1.38–4.10)	1.34 (0.77–2.31)
2	17615	0.49	4.71 (2.83–4.10)	2.10 (1.25–3.51)
3	13100	0.48	4.63 (2.74–7.83)	2.03 (1.20–3.45)
4	6899	0.61	5.86 (3.37–10.19)	2.40 (1.37–4.20)
5	1498	1.13	11.00 (5.66–21.39)	3.91 (2.00–7.67)

GFR, glomerular filtration rate; CI, confidence interval; OR, odds ratio; HDL, high-density lipoprotein.

**Table 3:** Univariate and multivariable association between individual components of metabolic syndrome and decreased GFR.

Variables	Univariate Models*		Multivariable Models+	
	OR (95%CI)	*P*-value	OR (95%CI)	*P*-value
Elevated blood pressure	1.37 (1.03–1.82)	0.028	1.34 (1.00–1.78)	0.047
Elevated triglyceride	1.43 (1.12–1.82)	0.005	1.14 (0.88–1.49)	0.319
Reduced HDL-cholesterol	1.93 (1.51–2.47)	<0. 001	1.88 (1.44–2.43)	<0.001
Elevated fasting glucose	1.51 (1.16–1.95)	0.002	1.42 (1.09–1.85)	0.009
Obesity	0.97 (0.76–1.23)	0.795	0.78 (0.61–1.00)	0.054

GFR, glomerular filtration rate; CI, confidence interval; OR, odds ratio.

*Univariate models were adjusted for age and gender.

+ Multivatiate models were adjusted for age, gender and all the five metabolic syndrome components.

### Association between the number of MetS components and decreased GFR

There seemed to be a biologic gradient between the number of MetS traits and the prevalence of decreased GFR, irrespective of age and gender (Table 2). However, in analyses stratified by the type of components of MetS (Table 4), the multivariate-adjusted ORs (95% CI) of decreased GFR were 1.70(1.11–2.60), 2.38(1.53–3.71), or 4.11(2.42–6.98) for those with 1, 2, or 3 essential components (versus zero), including high BP, low HDL-c, high FPG, respectively. Conversely, the corresponding multivariate-adjusted ORs (95% CI) of decreased GFR were 1.11(0.84–1.48) and 0.89(0.63–1.27) for those with 1 or 2 non-essential components (versus zero), including high TG and obesity. After randomly sampling 50% of cases from the established database, the models were tautologically reconstructed 20 times, and consistent results indicated that this result was robust and stable.

**Table 4:** Multivariable association between the number of critical or noncritical metabolic syndrome components and decreased GFR.

Variables	Multivariable*	
	OR (95%CI)	*P*-value
Critical group		
0 component (reference)	1.00	
1 component	1.70 (1.11–2.60)	0.014
2 components	2.38 (1.53–3.71)	<0.001
3 components	4.11 (2.42–6.98)	<0.001
Noncritical group		
0 component (reference)	1.00	
1 component	1.11 (0.84–1.48)	0.457
2 components	0.89 (0.63–1.27)	0.527

GFR, glomerular filtration rate; CI, confidence interval; OR, odds ratio.

Critical group included elevated blood pressure, reduced HDL cholesterol, or elevated plasma glucose. Noncritical group included elevated triglycerides, or obesity.

*Multivariable logistic regression models included critical group and noncritical group shown above and also adjusted for age and gender.

## Discussion

The prevalence of decreased GFR was 0.4% in the present study, lower than the prevalence raised by Zhang et al [16] in a cross-sectional survey of China. Age was recognized as an independent risk factor for nephritic disease [1], [17]. Tanaka et al [18] indicated that the prevalence of reduced GFR increased markedly in Japanese participants aged 60 years or older. Our results consistently supported evidence for the notion that age played a significant role in renal dysfunction.

Substantial epidemiological studies, such as cohort studies [19]–[21], cross-sectional studies [22], and meta-analysis [23], on the association of MetS with CKD have yielded broadly consistent results that MetS was an independent risk factor for CKD, regardless of age, gender, and other potential confounders. Moreover, on histopathology examination, Singh et al [7] found that there was a steeper decline in renal function over time in patients with MetS. As expected, a strong association of MetS with decreased GFR was established in the present study, with an estimated OR of 1.43(1.13–1.83) after adjustment for age and gender. A similar study [9] performed on 19,012 Chinese populations aged 35 to 74 years revealed that the OR of the elevated serum creatinine with MetS was 1.36 (95% CI 1.07–1.73). Collectively, the apparent concerted result further indicated that MetS was a significant effective modifier in decreased GFR. To find measurements for reduced GFR control in public health practice, our in-depth analysis of the relationship between reduced GFR and various elements of MetS showed that even mildly elevated BP (≥130/85 mm Hg), FPG levels (≥6.1 mmol/L), or reduced HDL-c levels (<1.04 mmol/L) contributed to decreased GFR. Furthermore, the PARP of elevated BP, reduced HDL-c, and elevated FPG for decreased GFR was 27.55%, 19.67% and 14.31%, respectively. These findings illustrated that regulating BP to normal range (<130/85 mm Hg) could circumvent 27.55% clustering of risk factors. In the same vein, regulating HDL-c and FPG to normal range (≥1.04 mmol/L and <6.1 mmol/L, separately) could prevent 19.67% and 14.31% clustering of risk factors.

Studies have yielded mixed results for the association between various fractions of MetS and CKD. Chen et al [24], for example, reported a positive association between elevated BP, reduced HDL, elevated TG and CKD in a cross-sectional study of 6,217 U.S. adults. A Southeast Asian cohort study [10] included 3,195 participants and revealed that only high BP related to the prevalence of CKD. Recently, obesity, as an emerging threat, has also played an important role in the CKD [25]–[27]. The continuing debate over the deeply controversial issue of the association between individual components of MetS and CKD or reduced GFR revealed our lacking recognition of dynamic response of elements of MetS along with the developing process of renal dysfunction. Thus, ingredients of MetS and the cumulative number of Mets should not be considered as risk factors for decreased GFR indiscriminately.

Numerous studies reported a biologic gradient between the number of MetS ingredients and CKD [18]–[22]. At first glance, the current study was concordant with previous studies. However, an intriguing finding was presented after dividing all five components of MetS into critical arm (including elevated BP, reduced HDL-c, or elevated FPG) and noncritical arm (including elevated TG or obesity). The risk of reduced GFR was proportional to the cumulative number of critical components of MetS. Conversely, null association was found between decreased GFR and the cumulative number of noncritical components of MetS. To further validate this result, 50% of cases were randomly sampled from the established database, and the models were repeatedly reconstructed 20 times. Consistent results supported a paradigm-shaking hypothesis that decreased GFR was associated with the cumulative number of critical elements of MetS but not the non-critical elements. The impact of the number of MetS component on prevalence of CKD should therefore be interpreted judiciously. Future studies, of course, are needed to validate this possibility.

There are several strengths to our study. First, a large sample of healthy participants and early stage of patients were called to overcome information bias and prevalence-incidence bias. Second, we provided new insights to calculate the PARP of critical components of MetS. This may greatly help to find potential implications for controlling reduced GFR in public health practice. Third, to circumvent confounders, various MetS components were divided into critical arm and noncritical arm, the study of which, to the extent of our knowledge, was rare in this area.

This study also has some limitations. First, our population consisted mostly of urban workers who underwent an annual health checkup with a low prevalence of CKD. This study was therefore not nationally representative or geographically diverse. A completely random large sample to satisfy this study would be prohibitively expensive and impractical. Our cross-sectional analysis of a non-probability sample of the Chinese population decreases the accuracy of the prevalence, but not the mechanism of the study. Second, a cross-sectional design in the present study prevented us from drawing any definite conclusion unless a prospective follow-up study is performed. Third, the models in the current study were only adjusted for age and gender. Other associated factors such as marital status, income should also regard as potential confounders. However, relevant information was not obtained in this retrospective survey. Fourth, BMI was used instead of waist circumference in the definition of MetS due to a lack of corresponding data. This lack of data could have led to miss-estimation of the prevalence of MetS. However, we believe part of these potential limitations should be consistently attenuated by the large sample size of the study.

In conclusion, critical components of MetS including elevated BP, reduced HDL-c, and elevated FPG were significantly associated with decreased GFR with a PARP of 27.55%, 19.67%, and 14.31%, respectively. In addition, the prevalence of decreased GFR is solely linked to the cumulative number of critical elements with a biologic gradient present, but not the non-critical elements. Thus, the impact of MetS, its elements, and its number of elements on the prevalence of CKD should therefore be interpreted judiciously. Our study, to some extent, may facilitate the decision-making process of the public health, enabling early and appropriate critical elements recognition and elimination.
